# Revisiting the pink‐red pigmented basidiomycete mirror yeast of the phyllosphere

**DOI:** 10.1002/mbo3.374

**Published:** 2016-05-10

**Authors:** Alec Cobban, Virginia P. Edgcomb, Gaëtan Burgaud, Daniel Repeta, Edward R. Leadbetter

**Affiliations:** ^1^ Department of Geology and GeophysicsWoods Hole Oceanographic InstitutionWoods HoleMassachusetts02543; ^2^ Université de BrestEA 3882Laboratoire Universitaire de Biodiversité et Ecologie MicrobienneESIABTechnopôle Brest‐IroisePlouzané29280France; ^3^ Department of Marine Chemistry and GeochemistryWoods Hole Oceanographic InstitutionWoods HoleMassachusetts02543

**Keywords:** Ballistospore, D1/D2, phylogeny, physiology, rRNA, *Sporobolomyces*

## Abstract

By taking advantage of the ballistoconidium‐forming capabilities of members of the genus *Sporobolomyces*, we recovered ten isolates from deciduous tree leaves collected from Vermont and Washington, USA. Analysis of the small subunit ribosomal RNA gene and the D1/D2 domain of the large subunit ribosomal RNA gene indicate that all isolates are closely related. Further analysis of their physiological attributes shows that all were similarly pigmented yeasts capable of growth under aerobic and microaerophilic conditions, all were tolerant of repeated freezing and thawing, minimally tolerant to elevated temperature and desiccation, and capable of growth in liquid or on solid media containing pectin or galacturonic acid. The scientific literature on ballistoconidium‐forming yeasts indicates that they are a polyphyletic group. Isolates of *Sporobolomyces* from two geographically separated sites show almost identical phenotypic and physiological characteristics and a monophyly with a broad group of differently named *Sporobolomyces*/*Sporidiobolus* species based on both small subunit ribosomal RNA (SSU rRNA) and D1/D2 domains of the LSU rRNA gene sequences.

## Introduction

Among the basidiomycete yeasts, three distinct groups are now recognized on the basis of molecular phylogenetic studies of 5S ribosomal RNA (Walker and Doolittle 1982), SSU rRNA genes (Sush and Sugiyama 1993), D1/D2 domains of LSU rRNA gene (Fell et al. 2000), ITS1 and ITS2 rDNA regions (Scorzetti et al. 2002) and multigene approaches (James et al. 2006). Those three major phylogenetic lineages, namely Pucciniomycotina, Ustilaginomycotina, and Agaricomycotina are also well supported by the ultrastructure of their septum and the biochemical composition of the cell wall (Van der Klei et al. 2011). The Pucciniomycotina subphylum is comprised of a wide diversity of yeast life forms, including the genus *Sporobolomyces* that can easily be discerned from many other yeasts by the formation of reproductive ballistoconidia and specific phenotypic features, such as, the absence of xylose in whole‐cell hydrolysates, the presence of Q‐10 or Q‐10(H_2_) as the major ubiquinone isoprenologue, positive diazonium blue B and urease reactions, and the inability to ferment sugars (Hamamoto and Nakase 2000). While the presence or absence of ballistoconidia has been used to differentiate *Sporobolomyces* from *Rhodotorula* and *Bullera* from *Cryptococcus*, molecular data revealed that ballistoconidium‐forming yeasts are closely related to non‐ballistoconidium‐forming yeasts (Fell et al. 2001 and van de Peer et al. 1992). In addition, the formation of ballistoconidia is a polyphyletic character (Nakase 2000) and also a character that appears to be easily lost. This may complicate identification, underscoring the importance of applying a polyphasic approach to yeast taxonomy, that is, combining analysis of morphological features, biochemical composition, and phylogeny.

Some basidiomycetous yeasts are microbial producers of natural pigments, for example, the carotenoid‐producing genera *Phaffia*,* Rhodotorula*,* Cryptococcus*, and *Sporobolomyces* (Manimala and Murugesan 2014). Carotenoids have antioxidant properties, and these pigments are thought to confer resistance to many stressors by protecting from photodamage, by peroxy radical‐scavenging and by singlet oxygen quenching (Frengova et al. 1994). *Sporobolomyces* species (*S. roseus* (now recognized as *Sporidiobolus pararoseus*), *S. salmonicolor* (now recognized as *Sporidiobolus salmonicolor*) and *S. patagonicus*) include producers of torulene, torularodine, *γ*‐ and *β*‐carotene (Mata‐Gómez et al. 2014).

The *Sporobolomyces* genus is polyphyletic with more than 50 accepted species belonging currently to four lineages, that is, the Microbotryomycetidae, Sporidiobolaceae, Agaricostilbomycetidae families and the Erythrobasidium clade (Hamamoto et al. 2011). Many of these species were isolated from dead leaves that, when glued to the inner surface of a Petri dish lid, form a mirror image of the leaf outline and its veins on the opposing agar surface, due to the ejection of ballistoconidia. Thus, *Sporobolomyces* has been dubbed as the “mirror‐picture” or “shadow” yeast due to this capacity to reproduce a mirror image of its growth on an opposing agar surface. This is a feature that also allows for easy isolation of *Sporobolomyces* species from plant leaves. Ballistoconidia are mitotically produced cells that are capable of forced ejection from the parent fungus. Such ballistoconidia are discharged when a liquid droplet, called a Buller's drop, grows at the base of a conidium, leading to an abrupt displacement of spore mass as the surface tension on the drop is broken.

The documented polyphyly of *Sporobolomyces* was inspiration to investigate the phenotypic characteristics and phylogenetic relatedness of isolates from two geographically separated locations. While descriptive, investigations into phenotypic characters and genetic diversity of new isolates within specific taxonomic groups of microorganisms (especially groups that exhibit polyphyly) is informative for highlighting situations where species‐level assignments within that group may require reevaluation in the future. Incremental knowledge of specific taxons such as the ballistoconidium‐forming yeasts enhances the ability of other researchers who work with these organisms to evaluate characteristics of their isolates and to understand the group as a whole. We took advantage of the ballistic capabilities of the *Sporobolomyces* ballistoconidium‐forming yeasts to collect ten isolates from plant leaves from Vermont and Washington, on the Western and Eastern coasts of the United States, respectively. Our aim was to apply physiological and genetic analyses to gage how closely related some isolates of *Sporobolomyces* would be from these two geographically distant locations. The D1/D2 domains of large subunit ribosomal RNA (LSU rRNA) genes are successfully used to resolve individual yeast species (Kurtzman and Robnett 1998), and were used recently to revise the taxonomy of yeasts (Fell et al. 2000; Kurtzman et al. 2011), to describe new species of yeasts (e.g., Burgaud et al. 2011) and specific yeast genera (e.g., Groenewald et al. 2011) and to investigate yeasts from environmental samples including deep‐sea environments (Burgaud et al. 2010; Nagahama et al. 2011). According to Kurtzman and Robnett (1998), species of basidiomycetes species do not diverge more than 1% in D1/D2 sequences. However, exceptions to this prediction have been identified (see Discussion in Kurtzman 2014). Bai et al. (2002) used the D1/D2 domain to reclassify more than 50 ballistoconidium‐forming yeast strains identified as *Sporobolomyces roseus*, illustrating the utility of this marker for analysis of isolates of this and closely related taxa. Phylogenetic analyses in this study were thus based on both the small subunit ribosomal RNA (SSU rRNA) gene and the D1/D2 domains of the LSU rRNA gene sequences, when possible.

## Materials and Methods

### Sample collection

Leaf samples were collected from living birch, maple, sycamore maple, ash, gingko, alder, aspen, red oak, hickory, and paper birch trees at sites in Pierce County, Washington, and Rutland County, Vermont. Leaves were picked at the base of the petiole, and placed into individual resealable polyethylene bags where they remained until processing could occur at Woods Hole Oceanographic Institution within 4 days of collection. Leaves were maintained between 20°C and 25°C prior to initiating cultures. Leaf collection took place between May and August 2014.

### Isolation of *Sporobolomyces*

Isolates of *Sporobolomyces* were obtained on a sterile medium containing 1.5% tap water augmented with 5% w/v dextrose, 1% w/v yeast extract and 1.5% of agar (YAD). This medium was also used for the culture of *Sporobolomyces* following isolation. Initial isolation was performed by attaching subsections of individual leaves to the inner surface of the lids of sterile Petri dishes containing the YAD medium using double‐sided adhesive tape. Plates were then incubated so that the leaf remained in a position above the agar surface without coming into physical contact with the medium. Incubations were carried out at laboratory benchtop conditions (aerobic, and approximately 21°C). Colonies of reddish‐pink *Sporobolomyces* were generally visible on the surface of the medium after 24–72 h. Colonies were then transferred to new plates of sterile YAD medium to obtain pure isolates.

### Tolerance to freezing

Cultures of *Sporobolomyces* freshly cultivated on YAD plates were incubated at −20°C for 24 h. After 24 h, some colonies were transferred to fresh medium and incubated under benchtop conditions. The original plates used for freezing cultures were reincubated at −20°C for a second 24‐h incubation, and after a total of 48 h at −20°C, the remaining colonies were inoculated onto fresh YAD plates and incubated at benchtop conditions.

### Tolerance to desiccation and elevated temperature

Petri dishes with a thin layer of sterile YAD medium were allowed to develop visible colonies of growth of each *Sporobolomyces* isolate. These plates with freshly transferred colonies were incubated at 37°C, inverted, with the Petri dish lid slightly ajar. After a period of 48 h, the medium was dried to a wafer‐thin layer, and separated from the bottom of the Petri dish. Some colonies on each plate were scraped from the surface of the dried medium, and this dried cell material was inoculated onto fresh media and visually checked for regrowth at benchtop conditions.

### Growth on solid versus liquid medium

*Sporobolomyces* isolates were inoculated onto solid media as described above, and into liquid medium using a sterile inoculating loop. Growth in liquid medium was monitored by observing an increase in turbidity and then reinoculating onto solid medium to confirm maintenance of a pure culture. All culturing for these tests were conducted at benchtop conditions.

### Tolerance to short‐term anoxia

Isolates on solid YAD medium were exposed to anaerobic conditions in a COY anaerobic chamber with an atmosphere of 90% nitrogen, 8% CO2, 2% hydrogen for 4 days, and then reinoculated on YAD medium to monitor growth.

### Survival following pasteurization

Liquid medium was preheated in a water bath at 70°C. Isolates were inoculated into this hot medium and allowed to incubate for 15 min. After 15 min at 70°C, tubes of liquid medium were removed to an ice bath. After cooling, samples were streaked on to YAD plates to monitor growth.

### Tests for growth on alternative carbon sources

All isolates from different geographic locations that were initially cultivated on YAD media were tested for their ability to grow on selected alternative carbon sources: pectin, a structural polysaccharide contained in the cell walls of terrestrial plants, and galacturonic acid, an oxidized form of D‐galactose, the primary component of pectin. Cultivation of *Sporobolomyces* isolates was attempted using sterilized tap water media with the following amendments: (1) 3% Pectin (from apple, Sigma‐Aldrich, St. Louis, MO, USA) + 1.5% Agar + 0.3% NaHCO_3_ (PA); (2) 3% Pectin; (3) 1% Galacturonic Acid + 0.1% KH_2_PO_4_ + 0.4% NaHCO_3_ (GA), and (4) 0.5% Galacturonic Acid + 0.05% KH_2_PO_4_ + 0.2% NaHCO_3_ + 1.5% Agar (GAA). Tests were processed under aerobic conditions on the benchtop, micro‐oxic conditions in a candle jar, and under anaerobic conditions in a COY hood with the same atmosphere described above.

For experiments testing the ability to grow under anaerobic conditions, we utilized the following 6 media: YAD, desiccated 1.5% agar medium with liquid yeast extract added, YAD with 30 *μ*L of methanol in the lid, PA, GAA, and YAD with Baker's yeast precultivated on the plate.

### Small subunit ribosomal RNA (SSU rRNA) and large subunit ribosomal RNA (LSU rRNA) gene amplification

The partial SSU rRNA gene sequence was amplified from each isolate using 50 *μ*L PCR reactions with a final concentrations of 1X Go Taq Flexi Buffer (Promega Corporation, Madison, WI, USA), 5 mmol/L MgCl_2_, 200umol/L dNTPs, 200 nmol/L 360F (5′‐CGGAGARGGMGCMTGAGA‐3′), and 200 nmol/L EukB (5′‐TGATCCTTCTGCAGGTTCACCTAC‐3′) and 1 unit Taq Polymerase. The PCR reaction consisted of 98°C for 30 sec, followed by 30 repetitions of 98°C for 10 sec, 55°C for 15 sec, and 72°C for 30 sec, followed by a final extension step of 72°C for 7 min. Timing did not allow for SSU rRNA amplifications for the Washington isolates. The D1/D2 region of the eukaryotic LSU rRNA (26S) gene was also amplified, using the same protocol as noted above, with the forward primer NL1 (5′‐TGCTGGAGCCATGGATC‐3′) and reverse primer NL4 (5′‐TAGATACATGGCGCAGTC‐3′). The PCR protocol utilized a 94°C 30 sec initial denaturing step, followed by 30 cycles: 94°C for 30 sec, 52°C for 30 sec, 72°C for 1 min, and a final extension temperature of 72°C for 7 min. PCR products were mixed with gel loading buffer (Ambion), in a 5 to 1 ratio, and were run on a 1% agarose gel with 1X Sybr Safe DNA Gel Stain (Thermo Fisher Scientific, Waltham, MA, USA) along with a 1 kb ladder (Invitrogen, Carlsbad, CA, USA) to confirm the correct size of the amplified PCR product.

### Sequencing of PCR products

PCR products from each isolate were precipitated using isopropanol and sent to Beckman Coulter Genomics (Danvers, MA) for Sanger sequencing. Sequence quality was visualized using Sequencher 5.3 (Gene Codes Corporation Ann Arbor, MI, USA). Primer sequence sites were removed and sequence ends were quality‐trimmed to remove ambiguous base calls using the software Sequencher version 5.2 (Gene Codes Corporation). An alignment of each gene target was generated using ClustalX 2.1 and an identity matrix calculated.

### Phylogenetic analyses

The 18S rRNA gene sequence alignment was manually refined within the ARB software (Ludwig et al. 2004). Phylogenetic analyses of 18S rRNA gene sequences and D1/D2 sequences of isolates incorporated sequences from all available closest relatives in public databases as determined by BLAST. Sequences of all closest relatives of isolates in D1/D2 and 18S rRNA analyses were type strains for those species. The type strains included in D1/D2 analyses were: (*Sporobolomyces roseus* CBS2646, *Sporobolomyces patagonicus* CBS9657, *Sporobolomyces salmoneus* CBS488, *Sporidiobolus pararoseus* CBS484, *Sporidiobolus ruberrimus* CBS7500 and CBS 7501, *Sporobolomyces phaffii* AS2.2137, *Sporobolomyces japonicas* CBS5744, *Sporobolomyces carnicolor* CBS4215, *Sporobolomyces blumeae* CBS9094, *Sporobolomyces salmonicolor* CBS490, *Sporobolomyces johnsonii* CBS5470) and for 18S rRNA was, *Sporobolomyces roseus* CBS486. Bootstrapping and determination of the best estimate of the ML tree topology for alignments of 18S rRNA genes and D1/D2 sequences for isolates were conducted with the Rapid Bootstrapping algorithm of RAxML (1000 bootstrap replicates) version 8.1.11 under the GTR model with maximum‐likelihood estimate of the alpha‐parameter. Analyses were run on the CIPRES portal (Miller et al. 2010). The 18S rRNA alignment retained 829 positions for phylogenetic analysis, and the D1/D2 alignment utilized 529 positions. Each alignment was also analyzed with Bayesian methods as implemented on the CIPRES portal. The program was set to operate with a gamma distribution and four Markov chain Monte Carlo runs starting from a random tree. A total of 4,000,000 generations were calculated with trees sampled every 100 generations and with a prior burn‐in of 100,000 generations. A majority rule consensus tree was constructed from 39,000 post‐burn‐in trees. Posterior probabilities correspond to the frequency at which a given node was found in the post‐burn‐in trees. Sequences from isolates have been deposited in GenBank (Accession numbers KT780706‐KT780721).

### Pigment analyses

All isolates had pigmentation that appeared identical, based on color. Carotenoid pigments of the Castleton VT ash leaf *Sporobolomyces* isolate were extracted by sonication in 1 mL methanol for ~ 1 h. Samples were protected from light in order to minimize isomerization and degradation of pigments. Suspensions of *Sporobolomyces* biomass and solvent were centrifuged (10 min, 2000 rpm), the supernate removed, and dried under a stream of ultra high purity (UHP) nitrogen. Pigments were dissolved in 100 *μ*L of methanol and separated by high pressure liquid chromatography (HPLC) fitted with a reverse phase column (Supelco; Ascentis C18, 150 mm × 2.1 mm; 3 *μ*m) and eluted at 0.3 mL/min with a linear gradient of 100% A to 100% B over 25 min followed by a 25 min hold at 100% B for initial analyses (where A is 80/20 (v/v) methanol/0.1% aqueous ammonium acetate, and B is 80/20 (v/v) methanol/acetone). For mass spectral characterization, we used the same column eluted with a linear gradient of 100% A to 100% B over 25 min followed by a 25 min hold at 100% B (where A is 80/20 (v/v) methanol/0.1% aqueous formic acid, and B is 80/20 (v/v) 0.1% methanolic formic acid/acetone). Absorption (250–800 nm) and mass (400–800 Da) spectra were collected using a diode array detector and linear quadrupole mass spectrometer using an APCI interface operating in both positive and negative modes (3000 V capillary voltage, corona current, 4 *μ*A (positive), 15 *μ*A (negative).

## Results

### Success of isolation and morphological features

**Figure 1:**
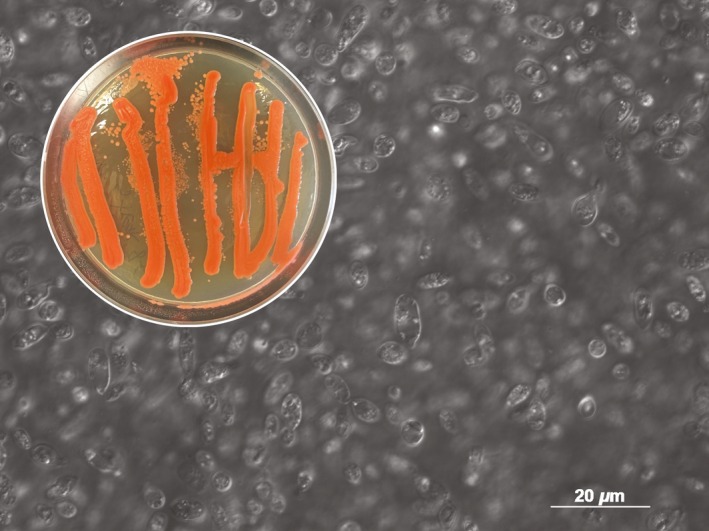
Light microscopy of common cell morphology for all *Sporobolomyces* isolates obtained in this study under differential interference contrast. Inset: pigmented growth characteristics common to all isolates.

Isolates of *Sporobolomyces* were obtained from ten different broad leaf sources, including seven leaves from Vermont (out of seven) and three (out of four) from Washington. In spite of being isolated from leaves originating from two distinct regions of the United States, all isolates displayed remarkably similar physical features among the morphological characteristics examined. All isolates were ~6–9 *μ*m long and ~3–4 *μ*m wide ellipsoidal cells forming salmon color pink pigmented smooth colonies. The ability to form ballistoconidia was observed for all isolates, as was a common red pigmentation of growth on solid media (Fig. 1). Light microscopy revealed morphological features typical of known *Sporobolomyces* under differential interference contrast (Fig. 1). Spatially separated growth was transferred to fresh media to confirm continued viability. It was possible to recultivate all isolates repeatedly on YAD media. The retention of ballistoconidia activity was confirmed after performing an inverted drop test, which involved affixing two Petri dishes one on top of the other, and each containing agar medium. The top plate had growth of the isolate, and the bottom plate was monitored for development of new growth originating from ballistospores from above. The physical and nutritional characteristics of all putative *Sporobolomyces* isolates were further characterized.

### Responses to freezing and desiccation

It was found that all isolates growing on YAD medium were still viable after three rounds of freezing at −20°C for 12–24 h, followed by thawing to room temperature. All isolates were determined to be either sensitive to desiccation or exposure to incubation temperature of 37°C. After cultures on YAD medium were placed in a 37°C incubator overnight until the medium appeared to be wafer‐like and devoid of moisture, cell material was scraped off and used as inoculum onto fresh YAD medium. Little to no growth was evident for all isolates, and whatever growth did appear, was not recultivable upon transfer.

### Nutritional flexibility and selected growth limitations of isolates

Sustained strong growth was observed for all isolates after repeated transfers on solid medium (1.5% agar) prepared with apple pectin as the only added nutrient as well as with 1% galacturonic acid (neutralized to pH 7.0). Baker's yeast was cultivated on YAD plates onto which the *Sporobolomyces* isolates were inoculated to test if each isolate might possibly utilize the ergosterol produced by the Baker's yeast under aerobic conditions (Valachovic et al. 2001) for subsequent growth under anaerobic conditions. Under aerobic conditions, growth of each isolate in the presence of Baker's yeast was not observably different than in the absence of the Baker's yeast. Under anaerobic conditions in a COY anaerobic chamber (atmosphere described above) no significant growth of any of the *Sporobolomyces* isolates was observed. This suggests that our isolates required oxygen for their growth. This was confirmed by no observable growth on either plain YAD medium or on 1.5% agar plus either apple pectin or galacturonic acid under anaerobic conditions (exposure was for four or more days in the anaerobic COY chamber). Anaerobiosis for 4 days did not, however, decrease the viability of the cultures. All were capable of growth after being returned to benchtop conditions. While our *Sporobolomyces* isolates were not capable of anaerobic growth under the conditions we tested, all were capable of sustained growth under micro‐oxic conditions (achieved through the use of a candle jar). After exposure to micro‐oxic conditions for 4 days, all isolates were viable and sustained growth was observed under benchtop conditions after reinoculation. The presence of methanol in the atmosphere appeared to inhibit growth of all isolates. All were determined to be capable of growing on both liquid and solid versions of all media tested, although growth in liquid medium was slower than on solid media (based on visual inspection of turbidity). All isolates grown in liquid media were capable of sustained growth after being reinoculated onto solid medium. All isolates were pasteurized at 70°C for 15 min and then removed to an ice bath. No growth was observed after inoculation of pasteurized isolates onto YAD plates.

### Phylogenetic relationships

**Figure 2:**
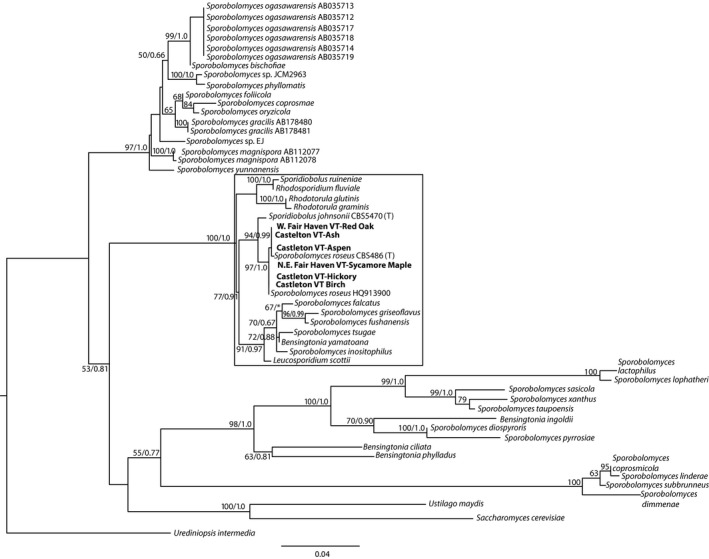
Maximum‐likelihood phylogenetic tree of 829 positions within the eukaryotic small subunit rRNA genes obtained from isolates. Sequences obtained from isolates are bolded. Bootstrapping and determination of the best estimate of the ML tree topology for this dataset was conducted with the Rapid Bootstrapping algorithm of RAxML version 8.1.11 under the GTR model with maximum‐likelihood estimate of the alpha‐parameter. Numbers at nodes present maximum‐likelihood bootstrap support above 50% and Bayesian posterior probabilities above 0.5, respectively.

**Figure 3:**
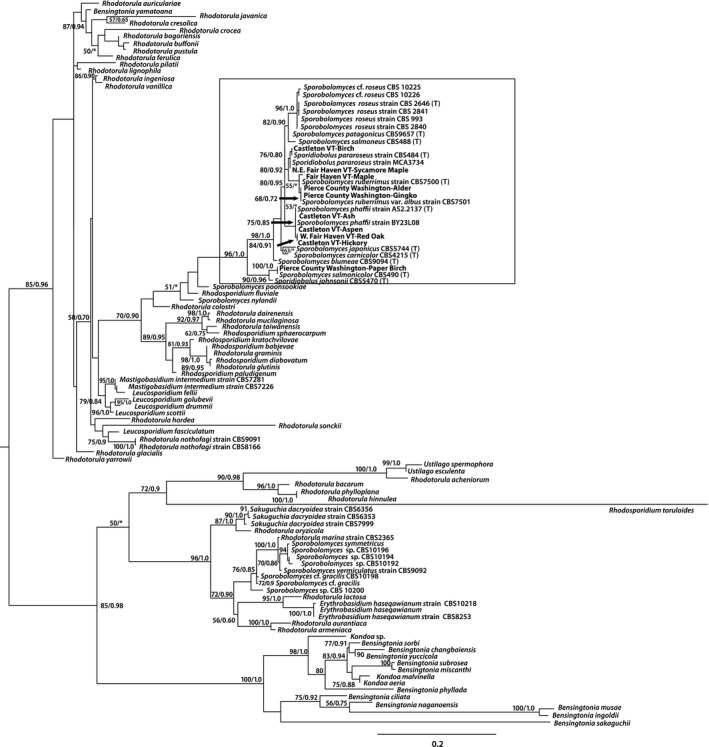
Maximum‐likelihood phylogenetic tree of 529 positions within the D1/D2 region of the eukaryotic LSU rRNA gene obtained from isolates. Sequences obtained from isolates are bolded. Bootstrapping and determination of the best estimate of the ML tree topology for this dataset was conducted with the Rapid Bootstrapping algorithm of RAxML version 8.1.11 under the GTR model with maximum‐likelihood estimate of the alpha‐parameter. Numbers at nodes present maximum‐likelihood bootstrap support above 50% and Bayesian posterior probabilities above 0.5, respectively.

An identity matrix of partial eukaryotic SSU rDNA sequences showed a minimum of 99% similarity between the different isolates, and the identity matrix based on the D1/D2 domain of LSU rDNA returned a minimum of 91% similarity, the most dissimilar isolate being the one isolated from Washington state from paper birch leaves. This isolate had between 91% and 93% D1/D2 sequence similarity with all other isolates. The next most dissimilar isolate was the isolate from Fair Haven VT maple, with between 96% and 98% sequence similarity to the other D1/D2 sequences. All other isolates were between 97% and 100% similar to one another based on their D1/D2 sequences. The partial SSU rRNA gene was successfully amplified from six of our Vermont isolates, including isolates from red oak and sycamore maple from Fair Haven VT, and ash, aspen, hickory, and birch from Castleton VT. The phylogenetic analysis under maximum‐likelihood criteria that included available SSU rDNA sequences of the closest relatives of these isolates in public databases reveals that the Vermont isolates all fell within one well‐supported (97% bootstrap support under maximum likelihood, and Bayesian support of 1) clade with *Sporobolomyces roseus* ×60181 (the SSU rDNA sequence for the type strain CBS486) and *Sporobolomyces roseus* HQ913900, originally isolated from a salt marsh in Nova Scotia, Canada, which has 99% sequence similarity to the type strain sequence (Fig. 2). These sequences were more loosely associated with those from a larger clade of *Sporobolomyces, Rhodosporidium, Leucosporidium*, and *Bensingtonia* genera. The phylogenetic analysis under maximum‐likelihood criteria that included available D1/D2 domain sequences of the closest relatives of these isolates, including type species of *Sporobolomyces/Sporidiobolus* reveals that all isolates fell within a well‐supported clade (96% bootstrap support under maximum likelihood, and Bayesian support of 1) that included *S. roseus, S. patagonicus, S. salmoneus, S. pararoseus, S. ruberrimus, S. phaffii, S. japonicus, S. carnicolor, S. blumeae, S. salmonicolor*, and *S. johnsonii* (Fig. 3).

### Pigment analyses

**Figure 4:**
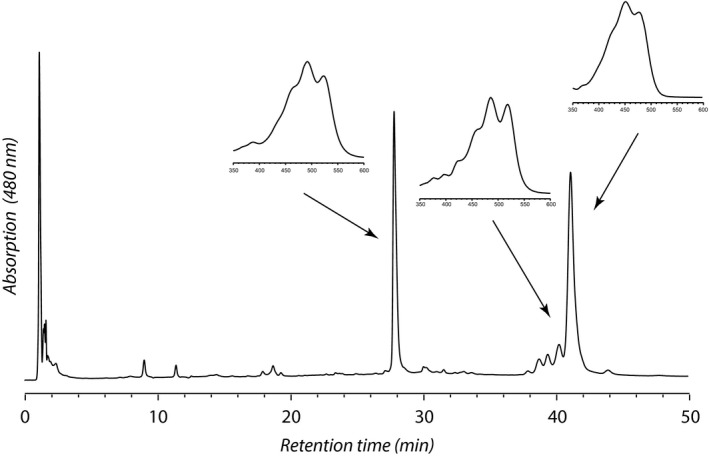
Chromatographic separation of carotenoids and porphyrins from the Castleton VT *Sporobolomyces* isolate. Conditions are given in text. Detection is at 480 nm. We observe three major carotenoid pigments, including torularhodin (27.8 min), torulene (40.2 min), and *β*‐carotene (41.0 min) as well as a number of *cis* isomers of torulene and *β*‐carotene eluting between 37 and 42 min. Insets show the on‐line visible absorption spectra for each pigment. Three additional pigments eluting at 9.5, 10.7, and 19.3 min were tentatively identified as porphyrins based on their visible spectra.

Two major and one minor carotenoid pigments from the Castleton Vermont ash isolate were separated and detected by HPLC. Of the two major peaks, the most polar peak (27.8 min) displayed a visible spectrum with adsorption maxima at 303, 468, 492, and 522 nm, in good agreement with published values for torularhodin (*λ*_max_ 465, 490, 519 nm; Moliné et al. 2012). We were not able to unambiguously assign a molecular ion to this compound under ESI‐ and APCI‐ conditions. The least polar peak (41.0 min) displayed adsorption maxima at 426(s), 450, and 476 nm and a molecular ion (M + 1) at m/z 537 Da, and was identified as *β*‐carotene. Immediately preceding the *β*‐carotene peak at 34.3 min, we detected a minor peak of torulene, with *λ*_max_ 457, 485, 518 nm and a molecular ion at m/z 535 Da (Fig. 4).

## Discussion

The genus *Sporobolomyces* encompasses several species commonly found in different habitats, with plant leaves as the most representative origin substrate. While numerous species have been isolated from dead leaves, few studies have compared ballistoconidium‐forming taxa from different geographical locations. Here, we aimed to isolate different *Sporobolomyces* from two geographically distant locations, one located on the eastern and one on the western coast of the United States and to examine physiological and phylogenetic variations in the isolates.

Previous findings indicated the polyphyly of *Sporobolomyces*, with species belonging to four lineages, that is, *Microbotryum, Sporidiobolus, Erythrobasidium*, and *Agaricostilbum* (Hamamoto et al. 2011). This suggests either a close relationship between *Sporobolomyces* and these other sister taxa, or the possibility that the taxonomic assignments of some sequences from GenBank may require reassessment. In a previous study of SSU rDNA gene sequences of *Sporobolomyces* and *Sporidiobolus* isolates, considerable morphological and phylogenetic diversity was observed between different *Sporobolomyces* species, calling into question the taxonomic classification of some species at the generic level (Hamamoto and Nakase 2000). It is interesting to note that although our sampling of *Sporobolomyces* species in these two geographically separated locations was not exhaustive, the sequences of our isolates fell within well‐supported clades based on SSU rDNA (Fig. 2) and LSU rDNA D1/D2 (Fig. 3) together with sequences of type strains of a subset of *Sporobolomyces*/*Sporidiobolus* species included in these analyses. Previous SSU rRNA phylogenies of basidiomycete yeasts place *Sporobolomyces* species within five major lineages within the Pucciniomycetes (formerly known as the Urediniomycetes), illustrating the polyphyletic nature of this genus (Hamamoto and Nakase 2000). The highly conserved nature of SSU rDNA can make it difficult to resolve phylogenetic relationships among closely related species. As a result, additional phylogenetic markers are routinely used for yeasts, including the D1/D2 domain of LSU rDNA (see above). The internal transcribed spacer (ITS) region has also been applied (Brookman et al. 2000; Tuckwell et al. 2005; Fliegerova et al. 2006), however, abundant intraindividual variations within the ITS make this region inappropriate for studies of yeast diversity (Eckart et al. 2010). We obtained D1/D2 sequences for isolates from birch, ash, aspen, and hickory from Castleton VT, from sycamore and red oak from Fair Haven VT, and from paper birch, alder, and gingko from Washington. Our SSU rDNA analysis places our isolates within a clade (maximum‐likelihood bootstrap support of 97% and Bayesian support of 1.0) that includes the sequence for *Sporobolomyces roseus* type strain (Fig. 2). Our D1/D2 phylogeny places our isolate sequences within a larger clade (maximum‐likelihood bootstrap support of 96% and Bayesian support of 1.0) that includes the sequence of the type species for *S. roseus*, but in addition, sequences of type species for *S. salmonicolor, S. blumeae, S. carnicolor, S. japonicus, S. phaffii, S. ruberrimus, S. pararoseus, S. salmoneus,* and *S. patagonicus*. Isolates from the same geographic location do not appear to be more closely related to one another than they are to isolates from the other, more distant location, although it is premature to draw conclusions from this observation given the small sample set from each site.

All isolates were successfully cultivated under aerobic and microaerophilic conditions on media containing pectin as the principal carbon source, as well as media containing galacturonic acid. All were tolerant of repeated freezing and thawing, were mildly tolerant of desiccation, and could grow in liquid or solid media. Although previous work has described similar isolates as members of differing genera, we suggest that there may be little genetic or physiological variation between isolates identified as *Sporobolomyces* versus other closely related genera. We observed highly similar morphological and physiological attributes of our isolates, and highly similar SSU rDNA (99–100%) and D1/D2 domains of LSU rDNA (97–100% for most isolates). Pigment composition identified in *Sporobolomyces* isolates shares major similarities (*β*‐carotene, torulene, and torularhodin) with pigment compositions identified previously in *Sporobolomyces roseus* and *Rhodotorula* yeasts (Davoli and Weber 2002; Moliné et al. 2012).

Microorganisms of the phyllosphere, including ballistoconidium‐forming yeasts, encounter different kinds of stress, including low water activity, fluctuating temperature, strong ultraviolet radiation, etc. The ability of *Sporobolomyces* to eject conidia into the air tends to explain why some strains have been isolated from the atmosphere, for example, *S. roseus* (Fell et al. 2002). Interestingly, the harsh conditions that can be encountered on plant leaves may also be experienced in clouds. Recent cloud microbiology analyses have revealed a high proportion of *Sporobolomyces* (Vaïtilingom et al. 2013), suggesting here the ability of ballistoconidia to survive in this specific “habitat.” Clouds may play a major role in the distribution of microorganisms over the globe. Therefore, our recovery of closely related *Sporobolomyces* species on leaves of different deciduous trees at locations on both the west and east coast of the United States that share almost identical phenotypic and physiological properties (among those examined here), is not surprising. If dispersed to geographically distant locations via the air, ballistoconidium‐forming yeasts have likely evolved a shared capacity to adapt to varied habitats.

## Conflict of Interest

The authors declare they have no conflicts of interest to report.
